# An anthropomorphic body phantom for the determination of calibration factor in radionuclide treatment dosimetry

**DOI:** 10.1093/rpd/ncad176

**Published:** 2023-06-17

**Authors:** Bilal Kovan, Bayram Demir, Emine Göknur Işık, Duygu Has Şimşek, Zeynep Gözde Özkan, Sekan Kuyumcu, Cüneyt Türkmen, Yasemin Şanlı

**Affiliations:** Istanbul Medical Faculty, Department of Nuclear Medicine, Istanbul University, Fatih 34080, Turkey; Science Faculty, Department of Physics, Istanbul University, Fatih34080, Turkey; Istanbul Medical Faculty, Department of Nuclear Medicine, Istanbul University, Fatih 34080, Turkey; Istanbul Medical Faculty, Department of Nuclear Medicine, Istanbul University, Fatih 34080, Turkey; Istanbul Medical Faculty, Department of Nuclear Medicine, Istanbul University, Fatih 34080, Turkey; Istanbul Medical Faculty, Department of Nuclear Medicine, Istanbul University, Fatih 34080, Turkey; Istanbul Medical Faculty, Department of Nuclear Medicine, Istanbul University, Fatih 34080, Turkey; Istanbul Medical Faculty, Department of Nuclear Medicine, Istanbul University, Fatih 34080, Turkey

## Abstract

The aim of this study is to create an inhomogeneous human-like phantom, whose attenuation and scattering effects are similar to the human body, as an alternative to the homogeneous phantoms traditionally used in calibration factor (CF) determination. The phantom was designed to include the thorax, abdomen and upper pelvis regions sized to represent a 75-kg male with a body mass index of 25. Measurements using Lu-177 with 50- and 100-mL lesion volumes were performed using inhomogeneous anthropomorphic body phantom (ABP) and homogeneous NEMA PET body phantom. There was a difference of 5.7% of Calibration Factor including attenuation and scatter effect between ABP and NEMA PET body phantom. Because it better reflects the attenuation and scatter effect, it is recommended to use a human-like inhomogeneous phantom for determination of CF instead of a homogeneous phantom.

## Introduction

Today, as a result of developing technology and researches, many diseases are successfully treated by using new radionuclides and pharmaceuticals. This approach, called radionuclide treatment, is an important part of targeted oncological treatments today and its use is becoming increasingly common^(1)^. Depending on the uptake mechanism and excretion in radionuclide therapy, along with the tumour tissue healthy organs are exposed to radiation dose as well. In terms of toxicity, it is important to keep the radiation exposure of healthy organs to a minimum level while treating the target tumour tissue^(2)^. Today, a patient-specific dosimetric approach has gained importance while empirical dose approaches are widely used in conventional radionuclide treatment^(3)^. Although the organ dose limits used in external beam radiotherapy are used as a guide in determining the critical organ dose limits in radionuclide treatment, studies have revealed that the dose limits in radionuclide treatments are quite different^(4)^. Therefore, determination of critical organ dose limits for radionuclide treatments has become essential for effective and safe treatment^(5–7)^. As of February 2018, within the scope of the European Union, “European Union Basic Safety Standards Directive”, it has become a legal requirement to perform dosimetry for all patients receiving radionuclide therapy^(8)^. The basis of these efforts is to reduce the uncertainties in the radionuclide dosimetry to at least the values in external RT.

Dosimetric calculations in radionuclide therapy are made by using quantitative imaging methods, and the dosimetry guideline has been published by the European Nuclear Medicine Society (EANM) Dosimetry Committee^(9, 10)^. One of the most important steps of dosimetry is the derivation of a calibration factor (CF) to be used in determining the amount of activity in the patient's body. To create a CF, a known amount of radioactivity is subjected to SPECT imaging by placing it in a medium (phantom) where the effect of scattering and attenuation is created. By comparing the counts of this known source with the counts obtained from the patient, the amount of radioactivity collected in the organ/tumor is determined. Then, by means of these counts, organ/tumor doses are calculated mathematically^(10)^. This factor is of great importance in treatment dosimetry and has a direct effect on determining the organ/tumor dose.

**Table 1:** The averages of HU values obtained from patients and used materials.

	Soft tissue	Bone	Fat	Lungs
Patients	+ 60 (±22)HU	+ 600 (±214.3)HU	−118 (±38.1)HU	−500 (±335.6)HU
Phantom	+ 80 (±7.6)HU	+ 680 (±73.4)HU	−135 (±34.0)HU	−485 (±36.5)HU

However, there are several effects that cause the reconstructed SPECT image to differ from the real activity distribution in the body, such as (i) dead time occurred in the detector at high count rates with high activity^(11)^, (ii) low signal-to-noise ratio (SNR) due to sort imaging time^(12)^, (iii) effects of spatial resolution on SPECT^(13)^, (iv) depth-dependent spatial resolution (DDSR) due to varying distance from the imaging system^(14)^, (v) partial volume effect (PVE) due to the limited spatial resolution of SPECT/CT system^(15)^, (vi) reconstruction algorithm effects^(16)^, (vii) scattering^(17)^ and (viii) attenuation effects caused by tissue^(18, 19)^. The effect of the phantom type, which reflects the scatter and attenuation effects mentioned in the last above two, on the determination of CK is a subject that is still being studied and there is no consensus^(20–26)^. Therefore, it is expected to be the closest environment to the patient in the environment that will have a scattering and attenuation effect. This will provide the most accurate scattering and attenuation correction using CT^(10, 20)^.

In order to minimise the uncertainty in CF, the aim of this study is to create an inhomogeneous human-like phantom, whose attenuation and scattering effects are similar to the human body, as an alternative to the homogeneous phantoms traditionally used in CF determination. The CF values obtained with the inhomogeneous anthropomorphic body phantom (ABP) developed for this purpose were compared with the values obtained with other inhomogeneous phantoms used in the literature.

## Material and method

### Construction of ABP

Hounsfield unit (HU), which gives density information of different organs and tissues in the human body, was detected by computed tomography (CT) imaging in the single photon emission computed tomography-computed tomography (SPECT-CT) device used in our clinic. For patient sampling, images of 10 patients (5 Female, 5 Male and 56 years old (±11) who had previously undergone CT in our clinic were evaluated retrospectively. HU values were determined by drawing region of interest (ROI) areas from lung, bone, fat and soft tissue areas from CT images. The mean values of HU obtained from the patients are given in Table 1. Materials such as polyurethane and epoxy were mixed at different percentages to create materials with different densities. CT scans of these materials were made, and HU values were determined. Based on the obtained HU results, the materials to be used for the soft tissue, bone, fat and lung tissues were determined. The averages of the materials used and the tissue HU values obtained from the patients are given in Table 1.

**Figure 1:**
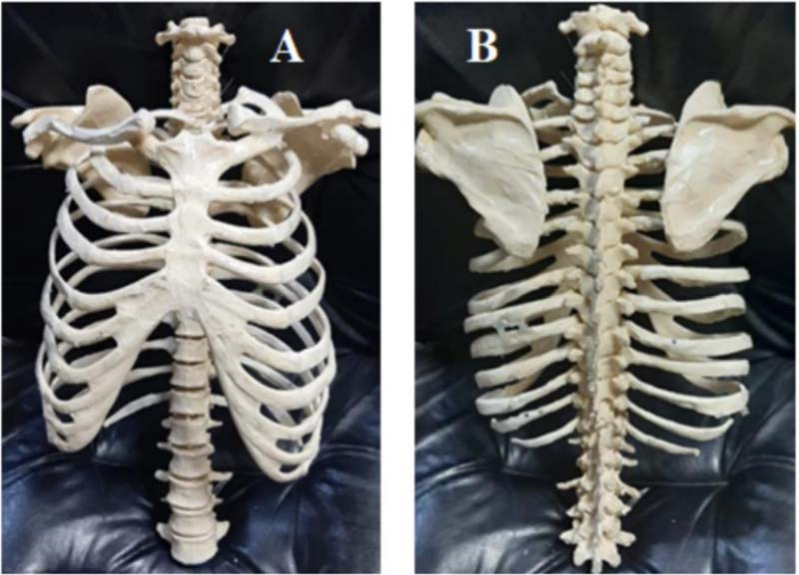
ABP skeletal system. (**A**) Anterior, (**B**) posterior.

The phantom was designed to include the thorax, abdomen and upper pelvis regions sized to represent a 75-kg male with a body mass index of 25. To create the skeletal system, negative molds were created for each bone structure with the help of mold silicone. The bones made with the help of negative molds were combined to form the skeletal system (Figure 1). Also, two lung-imitating sections, where the right lung volume is 1.3 lt and the left lung volume is 1.5 lt, were made into the phantom. The lung HU value was obtained by filling 66% Styrofoam foam and 34% water into the lung cavities.

**Figure 2:**
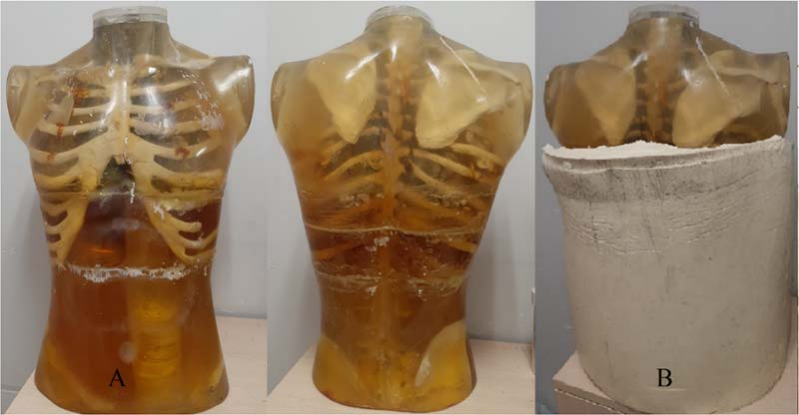
(**A**) ABP, (**B**) OABP.

Then the molds prepared for the lungs and the skeletal system were placed in the body mold. A plexiglas tube with an inner diameter of 5 cm and length of 60 cm was placed along midline (along craniocaudally axis) of the phantom for the lesions filled with radionuclide. Then, the phantom was formed by pouring the epoxy prepared to imitate the tissues into the body mold (Figure 2A). In addition, it was planned to construct an obese anthropomorphic body phantom (OABP) with 35 body mass index to control the body mass index-related change of CF in an inhomogeneous phantom. For this purpose, on top of the ABP, a removable layer of 7-cm-thick polyester material with a density equivalent to adipose tissue was produced (Figure 2B).

**Figure 3:**
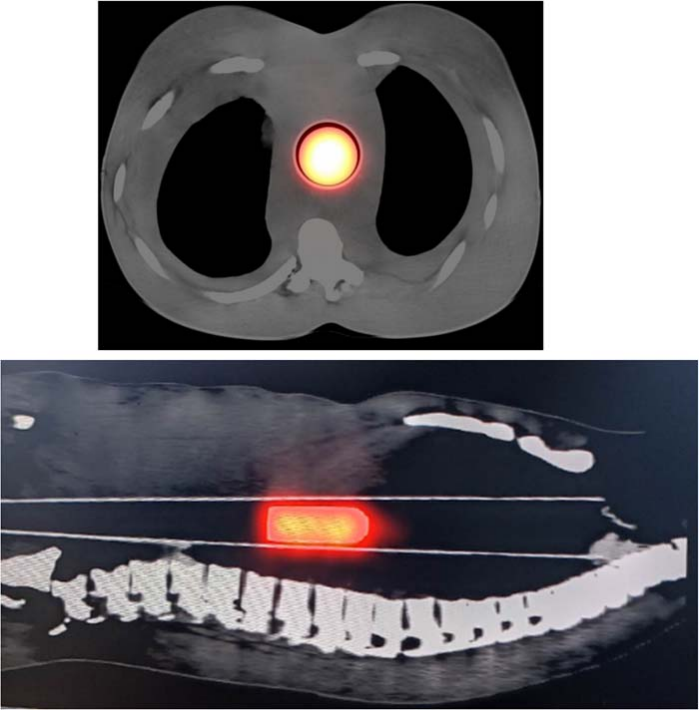
Central axial SPECT–CT slice (above) and *sagital* image of ABP with 100-mL lesion (with 4-cm diameter and 7-cm height) (below).

After the construction of the phantom, CT scan of the phantom was performed. A central axial slice form lungs and *sagital* image of whole phantom with lesion were given in Figure 3. Besides, all axial slices with thickness of 2 cm of phantom were given in the Supplementary material section of this study. HU values of all regions and their compatibility with human tissue densities were checked with value in Table 1.

### Determination of radioactive volume for CF measurements

One of the most important problems in the determination of CK is the effect of the volume of radioactive material on the CK value. A standard volume is not used in the literature to determine the CF. PVE, whose effects depend on the volume of radioactivity, is an important problem in quantitative radioisotope imaging^(15, 27)^. Wevrett et al. investigated the effects of PVE on SPECT counts with 16-, 8-, 4-, 2-, 1- and 0.5-mL spheres in their study. In various Gamma Cameras, they showed that the PVE effect was very dominant at volumes smaller than 8 mL, whereas the spheres with 16- and 8-mL volumes gave almost the same counts per MBq, so the PVE effect lost its importance after 16-mL volume. And, they measured a CF using spheres (max 37-mm diameter, 26.5-mL volume) of the NEMA/IEC phantom^(25)^.

In a study published in 2012 by Shcherbinin et al., they used a 70-mL plastic bottle for CF measurement^(28)^. In addition, there are studies in which CF calculations are made using 300-mL volume^(24)^, volumes of activity between 10 mL and 9 lt^(26)^, and volumes of activity ranging from 17 mm to 199 mL^(20)^.

Peters et al.^(29)^ reported that no PVE effect was observed after the 30.1-mm-diameter sphere in the Lu-177 study they conducted with Jaszczak phantom with six-spherical lesion (diameter of 9.9 (0.5 mL), 15.4 (2.0 mL), 19.8 (4.0 mL), 24.8 (8.0 mL), 31.3 (16.0 mL) and 60.0 mm (113 mL).

In the present study, to minimise the volume effect on the CK, a plastic vial with a volume of 50 mL (3-cm diameter, 7-cm length) and a plastic bottle with a volume of 100 mL (4-cm diameter, 8-cm length) was separately used to located in the internal space with 5-cm diameter created in ABP. This study aimed to measure CK in the most in-homogeneous body conditions. Thus, to simulation blood pool uptake, the plastic vial and plastic bottle are placed between the two lungs where the in-homogeneous environment is most concentrated. In this way, the effects of inhomogeneous environment on photon attenuation and photon scattering were maximised.

### Radionuclide and SPECT/CT imaging protocol

The radionuclide Lutetium-177 (177-Lu), which is widely used in systemic treatments, was used for the scans. Biodex Atomlab 500 (Biodex Medical Systems New York, NY, USA) dose calibrator certified by the accredited company was used for radionuclide dose measurements. GE Discovery NM 670 SPECT-CT (GE Medical Systems, Waukesha, WI, USA) device and medium-energy general-purpose collimator (MEGP) were used in the scans. In line with the EANM/MIRD Guidelines^(10, 30)^, during the SPECT acquisitions 60 projections, 20 second/projection, 128 × 128 matrix size, body contour on parameters were used.

The images were then reconstructed using Ordered Subset Expectation Maximisation (OSEM) with five iterations, 10 subsets and no post-filter as done in the study of Wevrett et al.^(25)^. During the CT acquisitions, 120-kV and 200-mAs parameters were used in line with the user manual recommendation of CT. Then, all phantom images were reconstructed without and with CT-based scatter and attenuation correction as described below.

### CT-based attenuation correction method

Electron emitting isotopes such as Lu-177 are generally used in radioisotope treatments. While these electrons are used to kill cancerous cells, internal dosimetry is performed with photons emitted immediately after electron emission. The purpose of the dosemeter is to determine the amount of radioactive material that the organ/tumor has accumulated. Then, the radiation dose received by the organ/tumor is calculated mathematically using this detected amount of radioactivity^(30)^. Gamma photons emitted from isotopes for dosimetry are used. However, the energies of the emitted photons in radionuclide treatment are generally in the range of 100–500 keV, and most of them cannot go out of the tissue as a result of photoelectric with the tissue and cannot be readout off by the detector. This phenomenon is known as the photon attenuation effect of tissue. The result of this events are a lower SPECT count than expected^(19)^.

Naturally, low SPECT counts also cause underestimation of organ/tumor doses. Thus, if the negative effects of attenuation on photon counts are corrected, the accuracy of the activity counts measured by SPECT is provided. These photons, which are absorbed in the tissue and cannot be counted by the detector, can be calculated with the help of CT and added to the primary SPECT counts^(18)^.

In the CT-based attenuation correction method, first, attenuation measurements obtained along all rays at all angles are created to produce a cross-sectional array of tissue attenuation coefficients (μ). Indeed, this resulting cross-sectional array of tissue attenuation coefficients (μ) an image of body attenuation. It is possible to show these data (μ) of various tissues on a standard grey scale. For this, the data (μ) are converted into CT numbers as HU by dividing to the attenuation coefficient of water using the following equation:


$$\mathrm{CT}\left(\mathrm{Number}\right)=\left(\frac{\mathrm{\mu} \mathrm{tissue}-\mathrm{\mu} \mathrm{water}}{\mathrm{\mu} \mathrm{water}}\right)\times1000$$

Now that we have a relation between μ and CT numbers. Thus, CT numbers can be used to correct for the attenuation effect of tissue. For the CT-based attenuation correction, CT scan is performed following the SPECT scan. In the CT scan, the device detects tissue densities and calculates the attenuation correction coefficient (μ) according to the gamma energy of the radionuclide used in SPECT acquisition, using the tissue density information it obtains. Then, it calculates the reductions that occur along the direction that the photon passes up to the gamma camera and makes attenuation correction by adding it to the counts.

Attenuation coefficients according to the CT number being less than or greater than 0 were calculated separately using the following equations and applied to the raw data by means of CT algorithm.

For CT numbers smaller than 0, because tissue is a mixture of water and air, and the attenuation coefficient of tissue ($\mathrm{\mu}\ \mathrm{tissue}$) at 208-keV energy can be calculated from the CT number by the following equation:


$${\begin{align*} & \mathrm{\mu} \left(\mathrm{tissue},208\ \mathrm{keV}\right) \\ &\kern-3pt=\!\left(\frac{\mathrm{CT}\left(\mathrm{Number}\right)\times\Big(\left(\mathrm{\mu} \left(\mathrm{water},208\ \mathrm{keV}\right)\!-\!\mathrm{\mu} \left(\mathrm{air},208\ \mathrm{keV}\right)\right)}{1000}\right) \end{align*}}$$

On the other hand, for CT numbers bigger than 0, the situation is getting a little more complicated because the tissue is a combination of water and bone. Therefore, in this situation, the attenuation coefficient of tissue ($\mathrm{\mu}\ \left(\mathrm{tissue}\right)$) at 208-keV energy can be calculated from the CT number by the following equation:


$${\begin{align*} &\mathrm{\mu} \left(\mathrm{tissue},208\ \mathrm{keV}\right)= \mathrm{\mu} \left(\mathrm{water},208\ \mathrm{keV}\right) \\ & \kern-3pt+\left(\frac{\begin{array}{@{}c@{}}\mathrm{CT}\left(\mathrm{Number}\right)\mathrm{\times}\left(\mathrm{\mu} \left(\mathrm{water},\mathrm{keVoff}\right)\ \times\ \right(\mathrm{\mu} \left(\mathrm{bone},208\ \mathrm{keV}\right) \\ -\!\mathrm{\mu} \left(\mathrm{water},208\ \mathrm{keV}\right)\end{array}}{1000\ \mathrm{\times}\ \Big(\mathrm{\mu} \left(\mathrm{bone},208\ \mathrm{keV}\right)-\mathrm{\mu} \left(\mathrm{water},208\ \mathrm{keV}\right)}\right) \end{align*}}$$

The study of James A. Patton and Timothy G. Turkington (2008) and Habib Zaidi and Bruce Hasegawa (2003) are recommended to the reader for further information about the CT-based correction method^(18, 19)^.

### CT-based triple energy windowscatter correction method

Photons emitted from radioisotopes in internal radioisotope treatment interact with atoms of the tissue in large amounts of Compton until they exit the tissue and reach the detectors. As a result of these interactions, they lose some of the emission photon energy and reach the detector as photons with lower energy and as coordinates different from the coordinates of the origin of emission by leaving their linear paths. In this process, of course, some scattered photons can overlap and reach the detectors with an energy higher than the photon emission energy, but again as coordinates different from their original coordinates. These scatter photons cause loss of image sharpness and removing these photons reached the detector by scattering too much from their path from SPECT counts will contribute to the improvement of the image^(19)^.

Compton scattering is one of the important problems of radionuclide imaging that has not yet been fully overcome. Although the studies on the correction method^(17, 31)^ are still continuing on this subject, the triple energy window (TEW) scatter correction technique method^(32)^, because of ease of implementation, good improvement of the image contrast and the low noise level, is proposed as most appropriate correction method^(33)^. It also proved to give accurate scatter estimates in phantoms^(32)^. TEW method by means of CT was also used for scattering correction in this study.

TEW scatter correction technique is a method to estimate the scatter within the photopeak window and it is based on using the information in two lower energy windows and one single calibration. In this method, the main window is located at the photopeak and the subwindows are located on left and right sides of the main window. The number of primary photons in the main window can be estimated using the area of a trapezoid obtained using photons of the two subwindows^(32)^.

If it assume that the width of the main window as Wm, and that of the subwindow as Ws, Counts (scatter) can be calculated from a trapezoidal area having a dimensions of a left height with C(left)/W(s), a right height with C(right)/W(s) and a base of W(m) using the below formula:


$$\begin{align*} \mathrm{Counts}\ \left(\mathrm{s}\mathrm{catter}\right)&=\left(\frac{\mathrm{Counts}\left(\mathrm{left}\right)}{\mathrm{W}\left(\mathrm{s}\right)\left(\mathrm{left}\right)}+\frac{\mathrm{Counts}\left(\mathrm{riht}\right)}{\mathrm{W}\left(\mathrm{s}\right)\left(\mathrm{right}\right)}\right)\\ &\quad \times\ \frac{\mathrm{W}\left(\mathrm{m}\right)}{2} \end{align*}$$

Then, the number of scatter corrected photons counts can be easily calculated by the following subtraction:


$$\mathrm{Counts}\left(\mathrm{corrected}\right)=\mathrm{Counts}\left(\mathrm{raw}\right)-\mathrm{Counts}\left(\mathrm{scatter}\right)$$

It is recommended to read the study of Hutton et al. (2011) for more detailed information about scatter correction methods^(31)^.

For the 208-keV main photopeak energy of Lu-177, two windows of 10% below (166.4–187.2 keV, centered at 176.8 keV) and above (228.8–249.6 keV, centered at 239.2 keV) were used. Scatter correction values were calculated using the following equation and applied to the raw data by means of the CT algorithm:


$$\begin{align*} &\mathrm{Counts}\ \left(\mathrm{scatter},208\ \mathrm{keV}\right)\\ &=\left(\frac{\mathrm{Counts}\left(176.8\ \mathrm{keV}\right)}{\mathrm{W}\left(176.8\ \mathrm{keV}\right)}+\frac{\mathrm{Counts}\left(239.2\ \mathrm{keV}\right)\ }{\mathrm{W}\left(239.2\ \mathrm{keV}\right)}\ \right) \\& \quad \times \frac{\mathrm{W}\left(208\ \mathrm{keV}\right)}{2} \end{align*}$$

**Figure 4:**
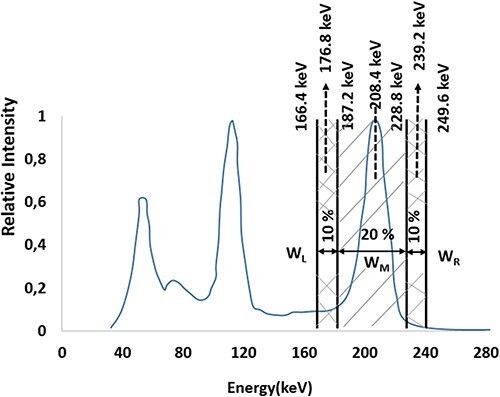
The application of the TEW method to the 208 keV (+/−10%) main energy peak of the Lu-177 used in this study.

In Figure 4, the main and subwindow widths of 208-keV energy peak of Lu-177 are shown based on the TEW method.

### Phantom measurements for CF and image analysis

**Figure 5:**
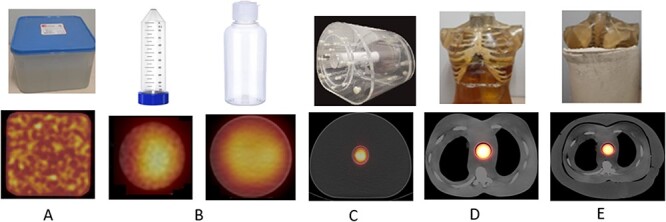
Five different phantoms used in the measurements. (A) 1500-mL water plastic container phantom, (**B**) 50-mL plastic vial (radius 3 cm and length 7 cm) and 100-mL plastic bottle (radius 4 cm and length 8 cm, (**C**) NPBP, (**D**) ABP, (**E**) OABP and their SCPET-CT images with 100-mL plastic bottle.

Measurements were done by five different geometry activity sources and different phantoms (Figure 5). (A) The SPECT–CT scanning was performed a 185-MBq Lu-177 activity by mixing with 1500-mL water (Plastic Container Phantom) after obtaining a homogeneous mixture. (B) For in-Air measurements, 177-Lu with 185-MBq activity was separately added in to a 50-mL water filled plastic vial and in to a 100-mL water filled plastic bottle. (C) Then, 50-mL plastic vial and 100-mL plastic bottle with the same activity (185 MBq) were separately placed into centre of the NPBP filled with water and scanning was repeated. (D) After then, 50-mL plastic vial and 100-mL plastic bottle were separately placed into the ABP to simulate blood pool uptake, and the scanning were repeated again. (E) Lastly, 50-mL plastic vial and 100-mL plastic bottle were separately placed into the OABP to simulate blood pool uptake of an obese patient, and the scanning were repeated again. To reduce statistical error, all acquisitions were repeated by using different activities of 92.5 MBq.

**Figure 6:**
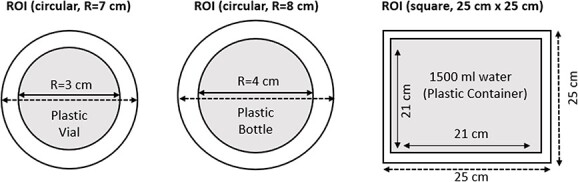
ROI plotting on SPECT axial images of phantom to determine counts. Left circular ROI (*R* = 7 cm) is for 50-mL vial (*R* = 3 cm), middle circular ROI (*R* = 8 cm) is for 100-mL plastic bottle (*R* = 4 cm) and right square ROI (21 cm × 21 cm) is for 1500-mL plastic container with a dimension of 21 cm × 21 cm on axial slice.

For determination of attenuation correction effects and scatter correction effects on the CF, the data obtained from phantom measurements were processed by CT-based attenuation correction and scatter correction. After processed, to determine the counts on the images, ROIs were drawn on axial phantom images. To include all counts to the results, ROIs with 7- and 8-cm diameter were separately drawn for the plastic vial with a 3-cm radius and for the plastic bottle with a 4-cm radius, respectively (Figure 6). For the plastic container phantom, ROIs were also drawn as a square with dimension of 25 cm × 25 cm wider than plastic container with dimension of 21 cm × 21 cm. ROI plots for phantoms are shown in Figure 6.

Then, volume calculations for measured activity were made with the help of these ROIs. After then, CF for all phantoms were separately calculated using the formula below for each situations of (Non Scatter Corrected + Non Attanuated Corrected; NSC + NAC), (with Scatter Corrected; SC), (with Attenuated Corrected; AC) and (with Scatter Corrected + with Attenuation Corrected; SC + AC) counts per MBq:


$$\begin{align*} &\mathrm{CF}\ \left(\mathrm{Counts}/\mathrm{MBq}\right) \\&=\frac{\mathrm{Total}\ \mathrm{Count}\ \mathrm{in}\ \mathrm{Plastic}\ \mathrm{Vial}\ \left(\mathrm{or}\ \mathrm{Bottle}\right)\ \left(\mathrm{Counts}\right)}{\mathrm{Activity}\ \mathrm{in}\ \mathrm{Plastic}\ \mathrm{Vial}\ \left(\mathrm{or}\ \mathrm{Bottle}\right)\ \left(\mathrm{MBq}\right)} \end{align*}$$

While making the calculations, the losses due to the physical half-life of the radionuclide were calculated and included in the result. The comparison of the CFs obtained from the measurements taken by five different geometries was made.

## Results

**Figure 7:**
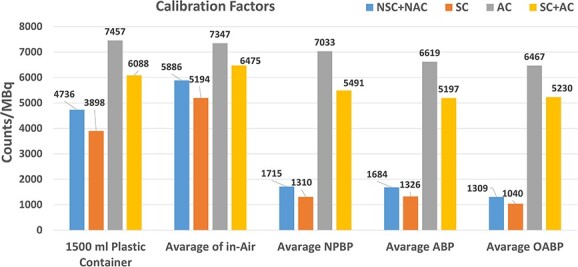
CF of phantoms for (Non Scatter Corrected + Non Attanuated Corrected; NSC + NAC), (with Scatter Corrected; SC), (with Attenuated Corrected; AC) and (with Scatter Corrected + Attenuation Corrected; SC + AC) as counts per MBq. The average values are the average of measurements with the 50- and 100-mL volumes.

The Corrected CF (SC + AC) obtained from the measurements taken by phantoms in five different geometries are given as Counts/MBq in Figure 7. Also, for all phantoms, CF for (Non Scatter Corrected + Non Attanuated Corrected as NSC + NAC), (with Scatter Corrected as SC), (with Attenuated Corrected as AC) counts per MBq were separately given in Figure 7. All values in the Figure 7 are the average of the measurement results taken with the 50-mL plastic vial and the 100-mL plastic bottle, except for 1500-mL plastic container.

In addition to Figure 7, in order to evaluate the volume effect in CF determination, CF values (only for Corrected CF (SC + AC) in Figure 7) measured with the 50-mL plastic vial, 100-mL plastic bottle and 1500-mL plastic container are given in Table 2 as normalised to ABP results.

**Table 2:** CF values (only for Corrected CF (SC + AC)) measured with 50-mL plastic vial, 100-mL plastic bottle and 1500-mL plastic container as normalised to ABP results.

Phantom		CF (normalise to ABP with 100 mL)	Average CF (50 and 100 mL)	Average CF (normalise to average ABP)
Plastic container (1500 mL)		1.175	1.175	1.171
In-Air	50-mL plastic vial	1.240	1.245	1.241
	100-mL plastic bottle	1.250		
NPBP	With 50-mL vial	1.061	1.060	1.057
	With 100-mL bottle	1.059		
ABP	With 50-mL vial	1.006	1.003	**1**
	With 100-mL bottle	**1**		
OABP	With 50-mL vial	1.012	1.010	1.007
	With 100-mL bottle	1.007		

## Discussion

Radiation has been used in cancer treatment for over a century. Over time, as the biological effects of radiation are understood more precisely, the range in treatment doses has begun to narrow. Although this increased the effectiveness of radiation therapy, it also reduced the side effects. Although external beams were used in radiation therapy at first, radionuclide treatments have been widely used in recent years. The use of external radiation therapy for many years has also increased the dosimetric experience, thus minimising dosimetric uncertainties. However, in radionuclide treatments compared with the external radiotherapy, the ratio of dosimetric uncertainties is more high due to the nature of the application (physiological reasons of patients)^(34)^ and the negativities such as the dosimetric method not being developed enough^(35, 36)^. Phantoms are of great importance in dosimetric calculations. The use of inhomogeneous phantoms imitating the human body in external radiotherapy dosimetric calculations has greatly increased the dosimetric accuracy^(37, 38)^. This is because inhomogeneous phantoms better reflect tissue radiation interactions such as scattering and attenuation effects. Thus, the use of accurate dosimetric measurement equipment has made a significant contribution here and the uncertainty in external beam radiotherapy has decreased to 2%^(39, 40)^. In radiation therapy, low doses may cause recurrence while high doses may cause irreversible side effects. It is reported that +/−5% accuracy is required in the delivery of absorbed dose to the target volume, even that in critical situations +/−2% may be required^(41)^. Therefore, uncertainties in radionuclide treatments should also decrease below 5% in the worst case^(39, 40)^. It is clear that the use of inhomogeneous phantoms in the emerging radionuclide therapy dosimetry will greatly reduce dosimetric uncertainties.

One of the studies about using a realistic human body phantom for radionuclide dosimetry is the study of Wevrett et al.^(26)^. In this study, an elliptical Jaszczak phantom was converted into a human-like environment to make it more realistic and used in the Lu177 CF measurements for an inter-comparison study. For this purpose, the spine part of phantom was filled with a bone equivalent solution to form bone density and lungs were filled with styrofoam mixed with water to form the lung density. However, bone structures, one of the most important sources of inhomogeneity in the real patient, were not formed in this modified phantom. By using this phantom with a 36 mm diameter spherical lesion which consist of two isolated concentric spheres, measurements were performed in 7 different centers of Europe. In the study conducted with this inhomogeneous phantom, it was revealed that there was no common dosimetric agreement between the users. The study reported that users generally tended to identify CF by an activity simply placed in the center of a Jaszczak phantom. Furthermore, it has been observed that there is a variation of up to 83% on the source activity determination among the centers methods. As a result of the study, the importance of determining CF with a common dosimetry method using a realistic human phantom in terms of radionuclide treatment dosimetry was emphasised.

Attenuation, which reduces the number of photons reaching the gamma camera, is an important problem whose negative effects on SPECT imaging need to be corrected. Habib Zaidi and Bruce Hasegawa^(19)^ showed that a very large amount of gamma photons emitted from nuclear medicine isotopes is absorbed in the tissue by the photoelectric effect. For example, only 10% of the photons with 140-keV energy at a depth of 15 cm can get out of the water while only 20% of the photons at 247 keV can get out of the water. The results of our study also show parallelism with this finding. As can be seen in Figure 7, the uncorrected counts coming from Lu-177 with 208-keV energy placed in the center of the ABP Phantom (NSC + NAC = 1684 Counts/MBq) are compared with the uncorrected in-air counts ((NSC + NAC = 5886 Counts/MBq), it is seen that the photons leaving the ABP phantom remain at about only 28%. The reason why our results are higher is that we used an inhomogeneous phantom containing lung tissue for the measurements while they calculated the absorption in homogeneous water.

On the other hand, if a comparison is made between NPBP (AC = 7033 Counts/MBq) and ABP (AC = 6619 Counts/MBq) in terms of photon attenuation, it is seen that there is a difference of 6.25%.

Another issue that has negative effects on SPECT imaging is photon scattering deal with in present study. Photons that are not completely absorbed by the photoelectric effect in the tissue may undergo Compton scattering by interacting with tissue electrons while leaving the tissue. Compton scattering is independent of Z and it decreases slowly with photon energy^(42)^. However, scattering is directly dependent on the thickness of the medium that the photon follows as it leaves the tissue. This is clearly seen in the results of our study. For example, when comparing the low-volume 1500-mL plastic container and the human-sized NPBP, ABP, OABP results in Figure 7; it is seen that 1500-mL plastic container used in present study does not adequately reflect neither the Compton scattering nor the attenuation that may occur in the patient. But anyways, although not as much as the photon attenuation effect, it is seen that there is a 1.2% difference when comparing photon scattering between NPBP (SC = 1310 Counts/MBq) and ABP (SC = 1326 Counts/MBq).

In another study of Wevrett et al.^(25)^, in which they determined CF using different gamma camera, they found differences from 6.6 to 34.8% in the measurements of CF with ^177^Lu radionuclide in the in-air, at the centre of the Jaszczak phantom and at different distances from the centre of Jaszczak phantom. They reported a 34% difference between results of a homogeneous elliptical Jaszczak phantom (8.9 cps/MBq) filled with water and results of in-air with sphere of 16 mL (12 cps/MBq). This difference, it can be seen as relatively in the last column of Table 2 of pthe resent study, was measured as 17.3% between measurements of homogeneous NPBP (1.058) and in-air (1.241). It is thought that the 16.7% difference between the homogeneous phantoms used in the studies is due to the difference between lesion volumes (16-mL volume versus 50/100-mL volumes) and phantom types (Jaszczak or NPBP). PVE resulted from volume is almost never detected in the present study because 50- and 100-mL volume results are very close to each other for all phantoms (Table 2).

In addition, in present study, it was determined that there was a difference of 5.7% between the corrected measurements results (SC + AC) performed with homogeneous NPBP and inhomogeneous ABP. Unlike the literature, it was demonstrated that the measurements performed with the ABP, which was used for the first time in this study, further improved the accuracy of CF values by 5.7%, since they reflect the actual attenuation and scattering. On the other hand, there was a 0.7% difference between the measurements made by using ABP and OABPs manufactured for patients of different weights. It shows that the CF value calculated with ABP can also be used for patients with different weights. CF is a gamma camera-specific value, so it should be used with an appropriate ABP when detecting CF in Nuclear Medicine clinics where dosimetric calculations will be made. It shows that the CF value calculated with ABP can also be used for patients with different weights. CF is a gamma camera-specific value^(25)^ and it should be used with an appropriate ABP when detecting CF in Nuclear Medicine clinics where dosimetric calculations will be made.

In this study, due to the nature of the phantom, an activity source to simulate different sized organs located off-centre could not be used. In future studies, phantoms with these features should be produced and measurements should be made.

## Conclusion

Today, with the spread of radionuclide therapy applications, internal dosimetric calculation methods are developing and specific critical organ doses for radionuclide therapies are redefined. A correct CF is one of the most important parameters for the accuracy of dosimetric calculations. The results of this study show that a using inhomogenously ABP for CF determination instead of a homogenously NPBP improves dosimetric calculations by 5.7%. Because it better reflects the attenuation and scatter effect, it is recommended to use a human-like inhomogeneous phantom for determination of CF instead of a homogeneous phantom.

## Conflict of Interest statement

None declared.

**Note:** This study was awarded “Prof Dr Suphi Artunkal Award” by the Turkish Society of Nuclear Medicine at the 34th National Nuclear Medicine Congress/Antalya/Turkiye.

## Data availability

Data will be made available on request.

## Supplementary Material

APPENDIX-Figures_ncad176
