# Supplementary figures and images for: An anthropomorphic body phantom for the determination of calibration factor in radionuclide treatment dosimetry
Source: Radiat Prot Dosimetry. 2023 Jun 17;199(12):1274–83. doi: 10.1093/rpd/ncad176 (PMC10372715; doi:10.1093/rpd/ncad176)

APPENDIX: Axial slices with thickness of 2 cm of ABP along craniocaudally axis.


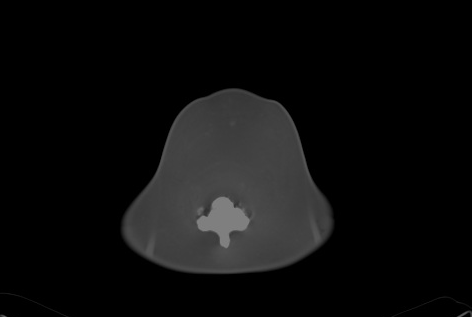


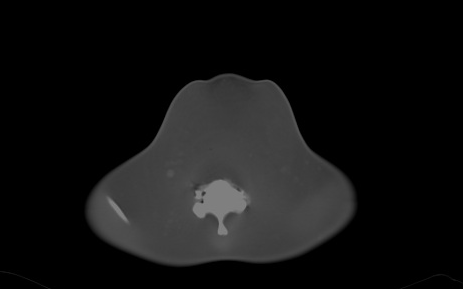


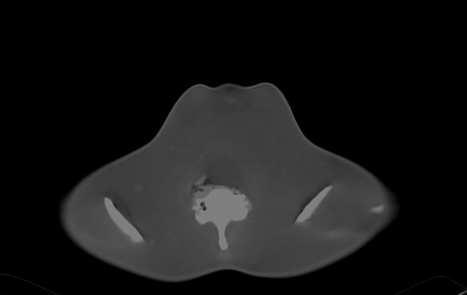


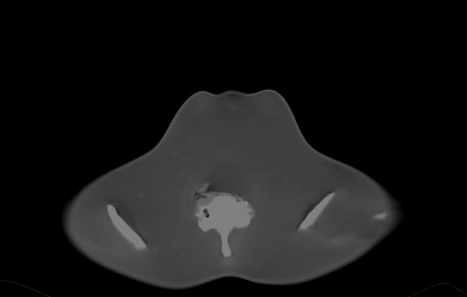


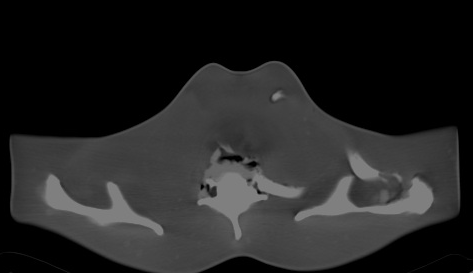


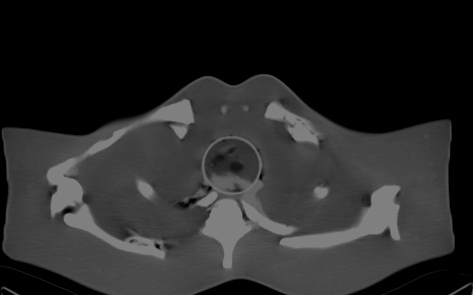


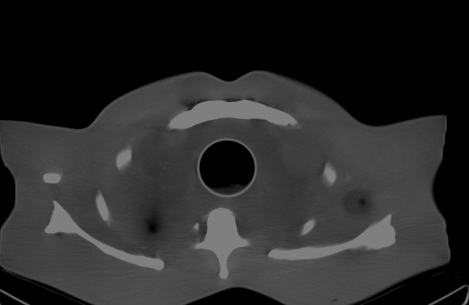


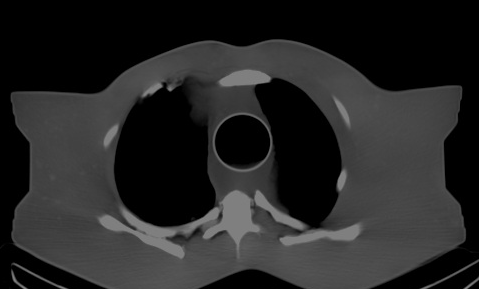


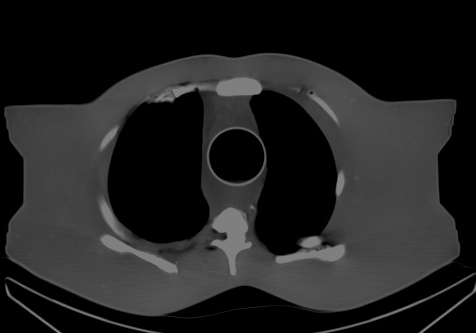


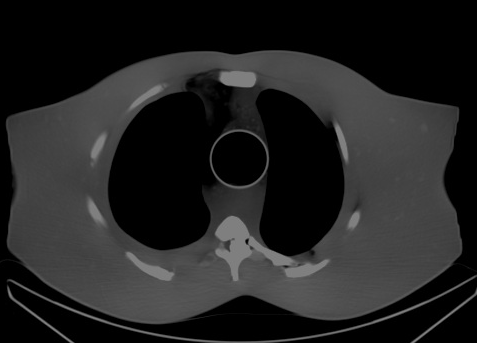


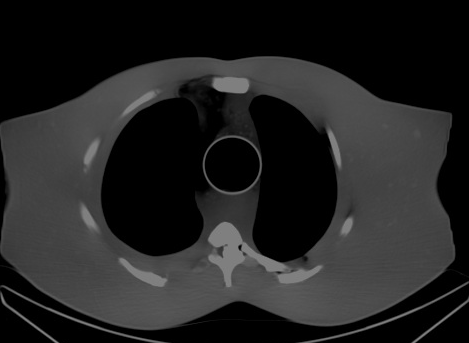


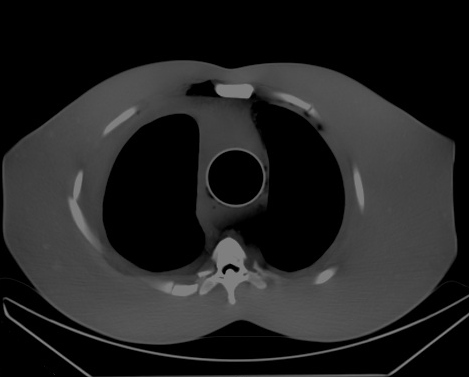


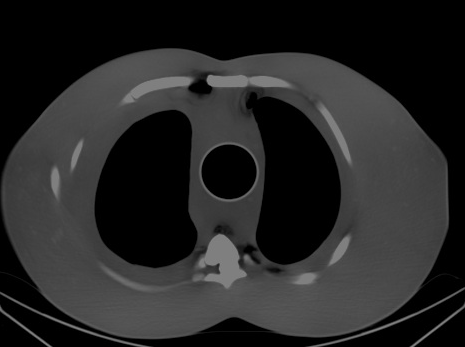


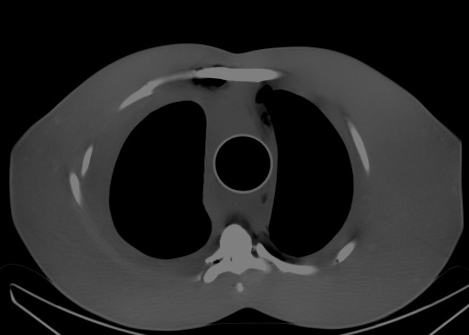


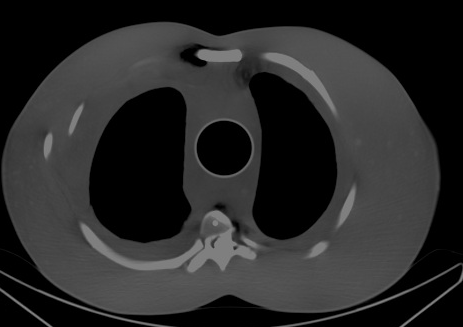


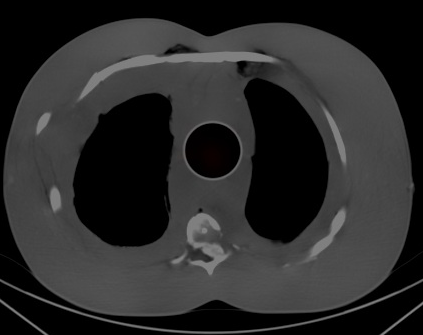


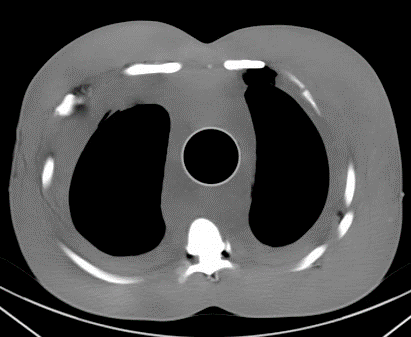


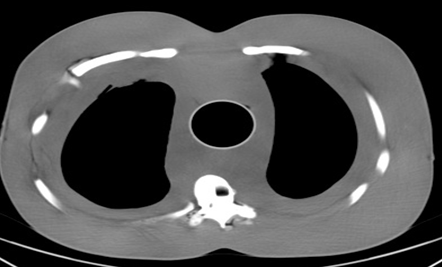


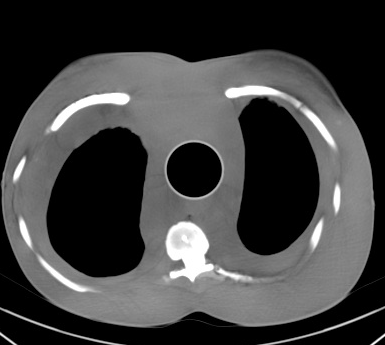


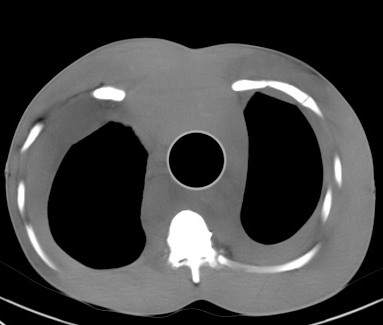


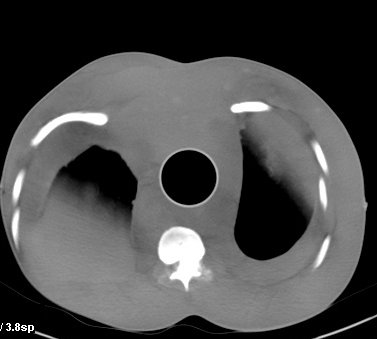


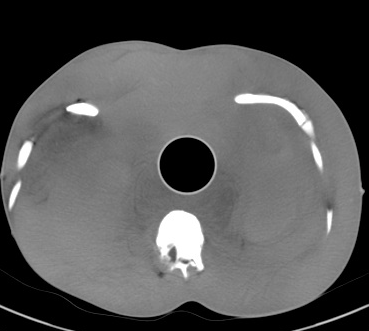


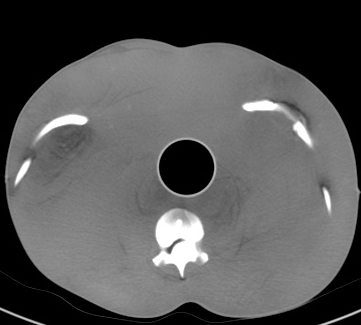


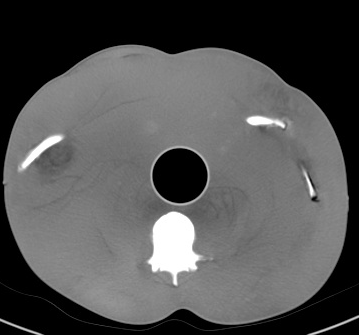


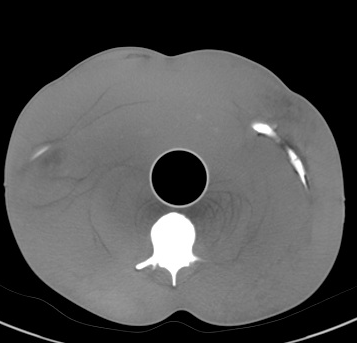


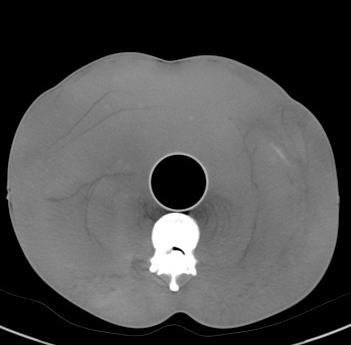


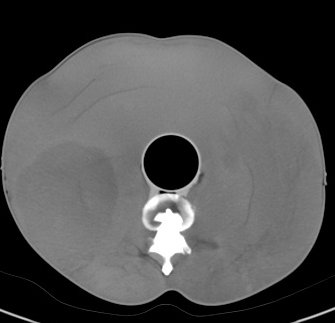


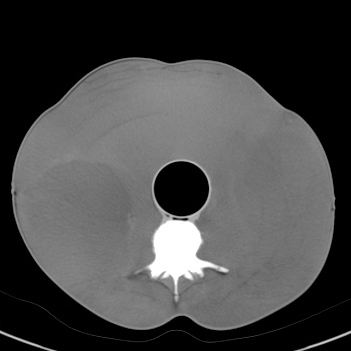


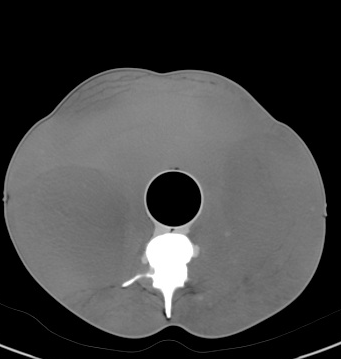


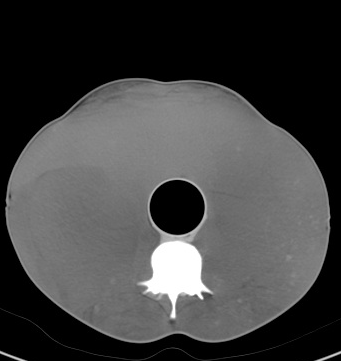


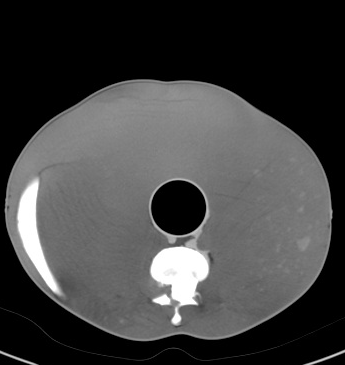


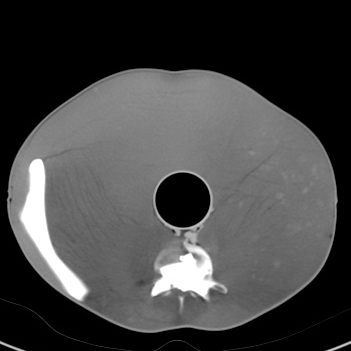


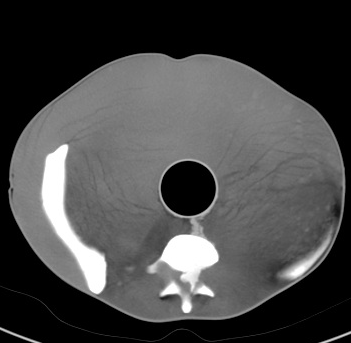


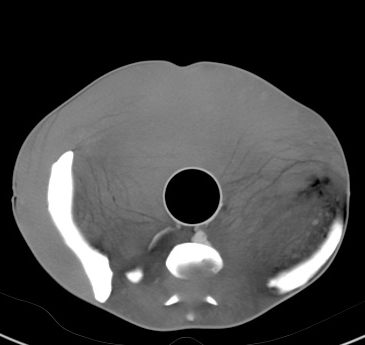


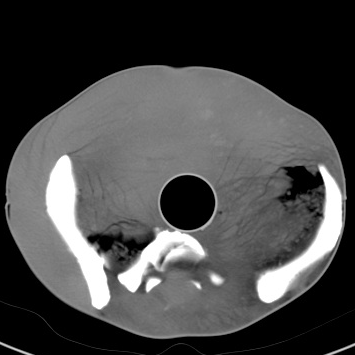


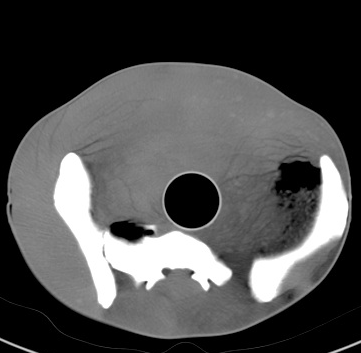

Supplement: APPENDIX-Figures_ncad176 [file appendix-figures_ncad176.zip › APPENDIX-Figures_ncad176.docx]
